# Comparison between 5 extractions methods in either plasma or serum to determine the optimal extraction and matrix combination for human metabolomics

**DOI:** 10.1186/s11658-023-00452-x

**Published:** 2023-05-20

**Authors:** Maryne Lepoittevin, Quentin Blancart-Remaury, Thomas Kerforne, Luc Pellerin, Thierry Hauet, Raphael Thuillier

**Affiliations:** 1 Inserm Unit IRMETIST, UMR U1313, University of Poitiers, Faculty of Medicine and Pharmacy, 86073 Poitiers, France; 2 IC2MP: Institut de Chimie des Milieux et Materiaux de Poitiers, Chemistry, France; 3 Cardio-Thoracic and Vascular Surgery Intensive Care Unit, Coordination of P.M.O. CHU Poitiers, 86021 Poitiers, France; 4 Biochemistry Department CHU Poitiers, 86021 Poitiers, France; 5 University Hospital Federation SUPPORT Tours Poitiers Limoges, 86021 Poitiers, France

**Keywords:** Metabolomic, Guideline, Extraction, Methods, Suitability

## Abstract

**Background:**

Although metabolomics continues to expand in many domains of research, methodological issues such as sample type, extraction and analytical protocols have not been standardized, impeding proper comparison between studies and future research.

**Methods:**

In the present study, five solvent-based and solid-phase extraction methods were investigated in both plasma and serum. All these extracts were analyzed using four liquid chromatography coupled with high resolution mass spectrometry (LC–MS) protocols, either in reversed or normal-phase and with both types of ionization. The performances of each method were compared according to putative metabolite coverage, method repeatability and also extraction parameters such as overlap, linearity and matrix effect; in both untargeted (global) and targeted approaches using fifty standard spiked analytes.

**Results:**

Our results verified the broad specificity and outstanding accuracy of solvent precipitation, namely methanol and methanol/acetonitrile. We also reveal high orthogonality between methanol-based methods and SPE, providing the possibility of increased metabolome coverage, however we highlight that such potential benefits must be weighed against time constrains, sample consumption and the risk of low reproducibility of SPE method. Furthermore, we highlighted the careful consideration about matrix choice. Plasma showed the most suitable in this metabolomics approach combined with methanol-based methods.

**Conclusions:**

Our work proposes to facilitate rational design of protocols towards standardization of these approaches to improve the impact of metabolomics research.

**Supplementary Information:**

The online version contains supplementary material available at 10.1186/s11658-023-00452-x.

## Background

Metabolomics is an emerging field and is broadly defined as the comprehensive measurement of all metabolites and low-molecular-weight molecules (≤ 1500 Da) in a biological specimen. Because metabolomics affords profiling of a much larger number of metabolites than are presently covered in standard clinical laboratory techniques, and hence offers the possibility for a comprehensive coverage of biological processes [1], it holds promise for tomorrow’s medicine [2–4]. Current metabolomic technologies are based on liquid chromatography coupled mass spectrometry (LC–MS) methods, capable of precise analyses of hundreds to thousands of metabolites in a single analytical technique through either open-ended (global) or targeted metabolomics [5, 6].

A range of biological samples (such as urine, blood, cerebrospinal fluid, saliva, etc.) can be utilized for metabolomics analysis. Nevertheless, blood, either plasma or serum, is the biofluid of choice for biological and clinical studies in general, particularly as regards metabolomics. Blood can be collected with little invasiveness and is rich in biological information. Blood derivatives contain metabolites secreted by different tissues in response to various physiological stimuli, conditions, or stressors. Consequently, serum and plasma are sensitive to health or diseased conditions, genetic variations, environmental factors, lifestyle, nutrition habits, and drugs, and they can provide important information at a systemic level.

As noted by Mannello et al. [7], using an incorrect matrix (e.g. plasma instead of serum) can result in a misdiagnosis. Therefore, the preanalytical phase is crucial for accurate metabolomics analysis.

It's important to understand the impact of using different blood derivatives on the metabolome in order to effectively translate metabolomics studies to the clinical level. Plasma and serum are both derived from whole blood, but they have undergone different biochemical processes. Serum is obtained from blood that has clotted, while plasma is obtained by adding an anticoagulant to the blood to prevent clotting, and then collecting the plasma that rises to the top. Several studies lead investigations on metabolomics differences between plasma and serum, however they were limited to targeted approaches including approximately 100 metabolites [8]. To examine the impact of these biological matrices in wide range of metabolites and classes of metabolites, we therefore led the investigation with untargeted approach.

For this study, we also investigated the sample preparation step, essential for obtaining reliable and meaningful results. Sample preparation is still an area of high importance when a LC–MS method is developed to assay biological samples. It is used in the “optimization” of a sample in order to remove proteins and interfering molecules from the matrix, thus preventing build-up on LC column which improves lifetime and significantly increases the number of detected metabolites. The goal of sample preparation is hence to ensure that the analytical method maintains robustness and consistency, as expected in any bioanalysis [9]. The difficulty is that metabolomics, unlike genomics and proteomics, aims at measuring molecules with disparate physical properties. Accordingly, comprehensive metabolomic technology platforms typically take the strategy of dividing the metabolome into subsets of metabolites—often based on compound polarity, or common functional groups—and devise specific sample preparation and analytical procedures optimized for each group [10]. The metabolome is therefore measured as a patchwork of results from different analytical methods. As an emerging field that has been fostered by steady development of analytical instrumentations with new capabilities every year, the methods used in metabolomics continue to evolve and improve. However, a consequence of metabolomics laboratories using multiple procedures that are potentially subject to frequent refinement is that individual laboratories tend to have unique methods and thus there are comparatively few standard operating procedures commonly adopted across laboratories. Although this diversity of technologies is linked to innovation in the field, it lends itself to potential challenges when comparing data between laboratories because of issues like differences in precision of measurement for select classes of metabolites or nonoverlapping metabolite coverage. In addition, the degree of certainty in metabolite identification can vary among methods, ranging from metabolite identities rigorously confirmed using authentic reference standards to putative identifications using reference databases for signals that remain as “unknowns.” Hence, the need for normalisation in metabolomics is essential to develop common guidelines [6].

There are many alternative methods to solve these problems. The most widely used protocol for metabolomics of plasma or serum is precipitation with miscible organic solvents. Methanol and/or acetonitrile are commonly used due to their high metabolite coverage and low cost. However, the broad specificity of these solvent-based precipitations results in highly complex samples that hinder the detection of low abundance metabolites. On the other hand, methods combining solvent extraction and solid-phase extraction to remove phospholipids are not as commonly used in global metabolomics because they are more selective compared to standard extraction methods. These solid-phase extraction (SPE) methods tend to reduce overall metabolite coverage, but they may improve data quality through increased repeatability and reduced matrix effects [11].

Few studies have explored this aspect. Most notably, Alshammari et al. demonstrated that methanol-based method possessed the best sensitivity and accuracy for mass spectrometry [12]; and Tulipani et al. showed that the conventional and hybrid techniques may beneficially cohabit untargeted metabolomics protocols [13]. These studies are crucial in the development of a metabolomics protocol, but none have compared standard precipitation methods to SPE in terms of overlap. Comparison between studies is not possible because of the different instruments and data processing techniques used. Additionally, most studies only examine metabolome coverage or extraction repeatability, and no study to date has evaluated extraction quality, matrix effects, linearity, and selectivity simultaneously in both biological matrices.

We hence proposed to compare five extractions methods for plasma *vs.* serum based on metabolomics analysis. The effects of three conventional solvent-based protein precipitation procedures (methanol, methanol–acetonitrile, and acetonitrile) and the combination of two solvents deproteinization with the removal of phospholipids (methanol-SPE, and acetonitrile-SPE) were evaluated. The performance of each method/matrix couple was compared in terms of extraction quality and efficiency, metabolomics coverage, precision/minimization of matrix effects, linearity, sensitivity and accuracy. Overlap was also evaluated through semi-targeted metabolomics approach (i.e., intermediate between untargeted (global) and targeted methodologies). The results revealed that the biological matrix choice and/or extraction method could greatly affect the future of a study and we propose a combination of extraction/matrices which offer the widest metabolome coverage.

## Methods

### Chemicals and materials

Optima™ LC/MS grade acetic acid, methanol, acetonitrile, water, and Pierce™ LC/MS grade formic acid were purchased from Fisher Scientific (Ottawa, USA). LC/MS grade formic acid, ammonium acetate, and ammonium formate were purchased from Merck (Germany, EU). Phree™ phospholipid removal tubes (1 mL) were purchased from Phenomenex (California, USA).

Labelled internal standards included succinic acid-2,3-13C2, l-tyrosine-(phenyl-3,5-d2), l-tryptophane-(indole-d5), l-phenylalanine-3,3-d2, l-leucine-5,5,5-d3 and d-glucose-13C6 purchased from Merck (Germany, EU); l-lactic acid-13C3 from Toronto Research Chemicals (Toronto, USA). Unlabelled-internal standards were purchased in the form of thirty-three metabolomics organic acids mix from Cambridge Isotope Laboratories (Massachusetts, USA). The listing of this mix is shown in Additional file 5: Table S1.

### Biological samples

Blood samples were pools of plasma and serum collected from seven healthy donors (approval number 20.10.05.42632): two women aged 21 and 28 years and five men aged 24, 26, 28, 29 and 40 years, at the university hospital of Poitiers, France. Samples were collected in BD vacutainer fluoride/oxalate tubes (grey cap) for plasma and silica (red cap) tubes for serum. Each plasma sample was centrifugated at 2000*g* for 20 min at 4 °C and then plasma was recovered, pooled, aliquoted, transferred into prelabelled cryovials, and stored at − 80 °C within 2 h of blood collection. Each serum sample was allowed to clot in an upright position for 30–60 min at room temperature and then was centrifuged at 2000*g* for 20 min at 4 °C. Serum was recovered, pooled, aliquoted, transferred into prelabelled cryovials, and stored at − 80 °C within 2 h of blood collection.

### Sample spiking

All chemicals were weighed individually using a micro-electronic balance from Fisher Scientific (Ottawa, CA, USA) with a precision of 0.0001 g. Stock solutions were created by dissolving the accurately weighed chemicals in the appropriate solvents, with specific concentrations for each analyte, as outlined in the Additional file 5: Table S1 and stored at − 80 °C prior to further use.

Five solvents, three for conventional precipitation (methanol, methanol/acetonitrile (1/1, v/v), and acetonitrile) and two for hybrid-SPE (0.1% formic acid/methanol, and 0.1% formic acid/acetonitrile) were prepared according to three methods: (i) unspiked; (ii) differently spiked with standard mix (MI) of seven isotope-labelled standards (ISTDs) at four final standard concentrations (5 μM, 10 μM, 25 μM and, 50 μM); and (iii) spiked with commercial isotope-unlabelled mix (MII), including thirty-three organic acids, at one final standard concentration (10 μM). The entire mixture was created by combining and diluting the relevant stock solutions with the solvents (Additional file 5: Table S1).

MI and MII standards were used for data quality control, to monitor mass accuracies, retention times, signal intensities, eventual drifts in instrumental sensitivity, and to evaluate the ability of each sample preparation method to detect differences among unspiked, spiked, and differently spiked biological samples.

### Sample extraction

**Fig. 1:**
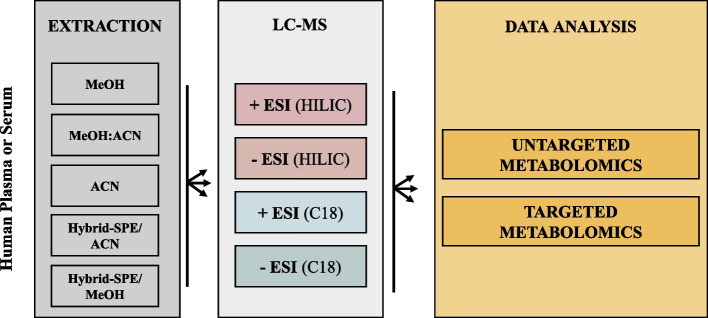
Overview of workflow comparing the five extraction methods in human plasma vs. serum. Five extraction methods, including three conventional (methanol, methanol/acetonitrile and acetonitrile) and two hybrid-SPE (with acetonitrile or methanol) methods, are performed in two different matrices (plasma vs. serum). All extracts are analysed according to (i) + ESI HILIC, (ii) − ESI HILIC, (iii) + ESI RP C18 and, (iv) − ESI RP C18. Finally, data analysis is evaluated across untargeted and targeted metabolomics approaches. *MeOH* methanol, *MeOH:ACN* methanol combined with acetonitrile, *ACN* acetonitrile, *SPE* solid preparation extraction, *ESI* electrospray ionisation, *RP* reverse phase, *LC–MS*: liquid chromatography coupled mass spectrometry

Five methods were used including both conventional protein precipitation and hybrid-SPE. Figure 1 presents an overview of the protocols with additional information provided in Additional file 1: Fig. S1). Each sample was subjected to the indicated high-throughput metabolite extraction procedures, in triplicate, to enable reproducibility measurements and to correct for systematic errors.

#### Protein precipitation

Preparation of pooled plasma and serum samples was carried out separately. In both cases, samples (50 μL) were thawed on ice and deproteinized by addition of spiked solvent (200 μL); the mixtures were vortexed, incubated at − 20 °C [1 h] and centrifuged (30 min, 15,000*g*), and the supernatants were collected. This solvent/sample ratio was chosen following a comparison between 1/3 and 1/4 ratios (Additional file 17: Table S13). Regarding the 1:5 ratio, it is commonly used for targeted lipidomics approaches [14]. However, for our project, this ratio was not compatible because it reduces the overall polarity of the solvent and greatly decreases the extraction of polar metabolites, which are our main study focus [15]. The three previously spiked solvents were methanol, methanol/acetonitrile (1/1, v/v), and acetonitrile at 4 °C.

#### Hybrid: SPE

Preparation of pooled plasma and serum samples was carried out separately. In both cases, sample preparation was carried out by solid-phase extraction, using phree™ phospholipid removal column. Samples (50 μL) were thawed on ice and dispensed into the Phree™ Tubes. The spiked acidic solvent (200 μL) (0.1% formic acid/methanol or 0.1% formic acid/acetonitrile) was then added into the Phree Tube to ensure precipitation. The Phree Tubes were centrifuged (15,000*g*, 4 °C) until filtrate is collected inside the collection tubes. The eluates were then collected and stored at − 80 °C before analysis procedure.

The protocols described above generated for each condition in triplicate a total of: (i) twelve plasma and serum extracts spiked with MI, (ii) three plasma and serum extracts spiked with MII, (iii) three unspiked plasma and serum extracts, and (iv) six neat solvents (blanks) spiked with MII. Blank samples were injected at the beginning and end of each batch to assess carry over and lack of contamination.

No external standards were added to the sample matrices after extraction. All samples were then stored at − 80 °C before analysis procedure.

### BCA assay and electrophoresis

Supernatants of plasma and serum extracts, previously obtained from the extraction methods, and blank extracts, corresponding to neat solvents, were analysed by BCA protein assay from Pierce Biotechnology (Rockford, USA) according to the manufacturer’s instruction, and SDS–PAGE was performed as previously described and protein load was visualised by stain free.

### LCMS analysis

All extracts (5 μL injection volume) were analysed on a Orbitrap Exploris 120 mass spectrometer interfaced with a Vanquish autosampler from ThermoFisher (Massachusetts, USA) in both positive and negative (ESI + and ESI −) modes. Samples were analysed using two chromatographic separations: (i) ACQUITY UPLC HSS T3, 1.8 μm, 100 mm × 2.1 mm column from Waters (Massachusetts, USA) in both positive and negative ionization mode and, (ii) ACCUCORE 150 Amide HILIC, 2.6 μm, 100 mm × 2.1 mm column from ThermoFisher Scientific (Massachusetts, USA) in both positive and negative ionization mode. The columns temperature was maintained constant at 35 °C in both ionization modes.

#### For ACCUCORE 150 Amide HILIC, 2.6 μm, 100 mm × 2.1 mm column

The mobile phase was composed of *A* = 5 mM ammonium formate and 0.1% formic acid in 95% acetonitrile and 5% water and *B* = 5 mM ammonium formate and 0.1% formic acid in 50% acetonitrile and 50% water for positive mode and *A* = 5 mM ammonium acetate and 0.1% acetic acid in 95% acetonitrile and 5% water and *B* = 5 mM ammonium acetate and 0.1% acetic acid in 50% acetonitrile and 50% water for negative mode. The gradient was applied: 1% B for 1 min, increased to 95% B over 9 min, held for 5 min, and then returned to 1% B to re-equilibrate the column for 6 min at a flow rate of 500 mL/min in both ionization modes.

#### For ACQUITY UPLC HSS T3, 1.8 μm, 100 mm × 2.1 mm column

The mobile phase was composed of A = 0.1% formic acid in 100% water and B = 0.1% formic acid in 95% acetonitrile and 5% water for both modes. The gradient was applied: 1% B for 0.5 min, increased to 99% B over 10.5 min, held for 1.5 min, and then returned to 1% B to re-equilibrate the column for 4.5 min at a flow rate of 300 mL/min in both ionization modes in both ionization modes.

In both case, ESI source conditions were set as follows: ion transfer tube 320 °C and vaporizer 400 °C, capillary voltage + 3200 V in positive mode and − 2700 V in negative mode.

The analysis was performed in the Full MS/ddMS^2^ (data-dependent MS^2^) mode, under which a Full MS scan event (SE1) was followed by a data- dependent scan with a fragmentation energy (SE2) applied. Ions of the second scan event then entered the HCD collision cell and were fragmented. The mass spectrometer acquired a Full MS scan at the resolution of 60,000. The automatic gain control (AGC) target (the number of ions to fill C-Trap) was set to 1.0e^6^ with a maximum injection time (IT) of 100 ms. The scan range of the Full MS scan was from *m/z* 100 to 1500. For the dd-MS^2^, the mass resolution was 16,000 FWHM with AGC target at 2.0e^5^, a maximum IT of 50 ms, and an isolation window of *m/z* 1.5.

### Data processing and analysis

Retention time, m/z (window) used for detection, limit of detection of the compounds are provided in the Additional file 15: Tables S11 and Additional file 18: Table S14. Example of chromatograms (highlighting the differences in the sample preparation protocols are also provided in the Additional file 19: Table S15. The chromatograms were obtained using the Freestyle software (Thermo Fisher Scientific).

Raw instrument data (.raw) were exported to Compound Discoverer 3.3 (CD3.3). Full workflow was optimised for deconvolution, alignment, refinement, compound detection and identification with online databases. Full MS/ddMS2 approach was allowed to detect and identify the added (un-)labelled internal standards with search mass list node [16]. A compound expected table containing mass, name, and molecular structure of standards was created and imported in CD3.3 (Additional file 6: Table S2). The search mass lists node searched compound expected table for masses that matched the detected compounds and thus created the mass list search results table. All detailed settings are provided in Additional file 2: Figure S2.

Data matrices from CD3.3 were subsequently exported as a.csv file including name and/or formula estimated, peak area, m/z, retention time and molecular weight estimated. For data analysis, all calculations were performed using Microsoft Excel 2010 and graphical generation using the R programming environment (RStudio Team, USA).

### Method validation

The method was validated with respect to the quality, linearity and sensitivity, accuracy and precision, recovery and matrix effects using FDA recommendations.

#### Quality

The quality was evaluated by measuring protein and biologically meaningless noise levels for each extraction method. The biologically meaningless noise level was defined as “unknown compounds” containing contaminants (chemical noise), artifacts (informatic noise), redundant signals (isotopes, adducts, in-source fragments, oligomers) and background compounds. Most of “unknown compounds” were controlled by parameters of CD3.3 software. Background compounds were calculated using the formula:

$${\text{Peak Area}}_{{{\text{Sample}}}} {\text{/Peak Area}}_{{{\text{Blank}}}} { < 5}{\text{.}}$$

#### Accuracy and precision

To assess accuracy and precision, measurements of three different concentration levels (low, medium, and high) were taken in triplicate for each labeled analyte and for all potential metabolites. The measured concentrations were then used to calculate the relative standard deviation (RSD), which was expressed as a percentage.

$${\text{RSD\% = }}\left[ {{\text{Standard Deviation }}\left( {{\text{SD}}} \right){\text{/Mean Peak Area}}} \right] \times {\text{ 100\% }}{.}$$

The acceptance for RSD in metabolomics is less than 30% (or 0.3) [17].

#### Linearity and sensitivity

The calibration curves were constructed by plotting peak area of the labelled analyte (*Y*-axis) against its known concentrations (*X*-axis), using three replicates per calibration point. The regression equations were described as *Y* = *a X* + *b*, and the linearities were assessed by the coefficients of determination (*r*^2^).

The detection limit (LOD) was determined as the lowest concentration that gives a signal-to-noise ratio of at least threefold (*S*/*N* > 3). The lower limit of quantitation (LLOQ) was defined as the lowest concentration of the calibration curve that gives a signal-to-noise ratio of at least tenfold (*S*/*N* > 10).

#### Matrix effect

The matrix effect (i.e., suppression and enhancement of metabolite signal in [M − H]^−^ or [M + H]^+^) was evaluated by analyzing the responses of label analytes prepared in solvent matrix and in extracted blood matrix at the same concentration. The value of matrix effect (ME) can be calculated from the following Eq. (18):

$${\text{Matrix Effect \% = [(Peak Area}}_{{\text{Post Extraction Spiked Matrix}}} {\text{{-} Peak Area}}_{{\text{Post Extraction Unspiked Matrix}}} {\text{)/Peak Area}}_{{{\text{Solvent}}}} {]}\; \times {\text{ 100\% }}{.}$$

Matrix effect in the range of 0.80 < ME < 1.20 (or 80% < ME < 120%), were considered as negligible and labelled as “no matrix effect”, since it is the common variability accepted in bioanalysis [19]. ME values below 0.80 (or 80%) were considered as ion suppression, and those above 1.20 (or 120%) as ion enhancement.

## Results

We compared the performance of five extraction methods for plasma and serum, using two complementary approaches: untargeted and targeted metabolomics.

### Untargeted (global) metabolomics analysis

#### Deproteination

Deproteination performances for the five extraction methods were evaluated for both matrices using BCA assay and electrophoresis (Additional file 7: Table S3; Additional file 3: Fig. S3). The results of total plasma/serum concentration proteins (µg/ml), after deproteinization are presented in Additional file 7: Table S3. In blanks, the analysis demonstrated the presence of minimal background noise (< 60 µg/mL), which is in accordance to the manufacturer’s specifications. For extracted plasma and serum samples, most extractions methods appeared to properly carry out protein removal. Considering the total protein concentration in plasma or serum approximately 70,000 µg/mL [20], we evaluated the ratio of remaining proteins to total proteins (Additional file 8: Table S4). This ratio was around 0.1–4.1% for all extraction conditions in plasma and serum. It should be noted that in hybrid-SPE with methanol, the protein values were tenfold higher than other extractions. A similar conclusion was reached by SDS-PAGE analysis, with important protein bands observed in the case of the hybrid-SPE extraction with methanol for plasma and serum (Additional file 3: Fig. S3).

#### Metabolite coverage

**Fig. 2:**
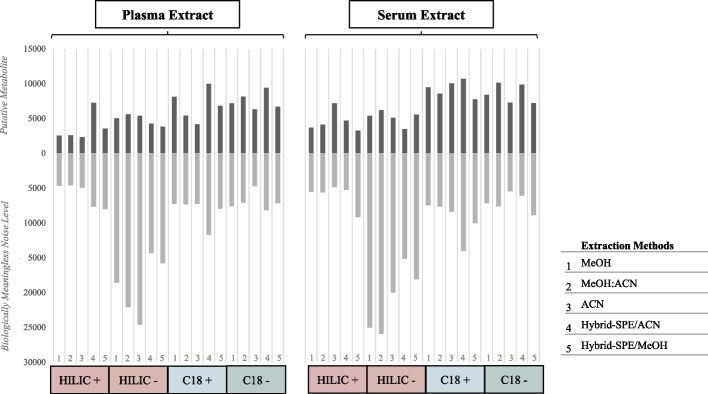
Coverage and quality analysis of plasma *vs.* serum through different extraction methods. The putative metabolite and « biologically unmeaningful noise level» peaks are analyzed following four LC–MS conditions: (i) + ESI HILIC, (ii) − ESI HILIC, (iii) + ESI RP C18 and, (iv) − ESI RP C18 within each extraction methods for plasma and serum. For total number of putative metabolites detected is analyzed after removal of features present in blank extracts.1: methanol extraction methods; 2: methanol:acetonitrile extraction methods; 3: acetonitrile extraction methods; 4: hybrid-SPE extraction method with acetonitrile; 5: hybrid-SPE extraction method with methanol

Figure 2 summarizes the putative metabolite coverage and “biologically meaningless noise level” of all extraction methods tested. After extractions, a combination of C18 reversed-phase (RP) chromatography and hydrophilic interaction chromatography (HILIC) is used in parallel to achieve comprehensive coverage of non-polar and polar metabolites, respectively. The results indicated a similar overall trend for plasma and serum. The number of detected putative metabolites fluctuated around 4500 in both + ESI and − ESI HILIC analysis while approximately 30% more putative metabolites were detected in both + ESI and − ESI RP analysis. Unexpectedly, “biologically meaningless noise level” was largely increasing in − ESI HILIC analysis, reaching values of over 25,000 signals in conventional methods for both plasma and serum.

#### Repeatability

Table 1 displays the repeatability of signals by presenting the mean RSD across all extraction methods for each LC–MS analysis protocol. Methanol and methanol–acetonitrile extractions for plasma showed the best repeatability among all other extraction methods, regardless of the LC–MS method used. When all four LC–MS methods were combined, the global mean RSD was below the acceptable limit. In the case of serum, only methanol extraction showed acceptable repeatability for global metabolomics. All the other extraction methods demonstrated very low repeatability, independently of LC–MS analysis (Table 1).

**Table 1:** Repeatability of extraction methods in plasma and serum assessed by global metabolomics analysis

		Mean RSD % (HILIC +)	Mean RSD % (HILIC −)	Mean RSD % (C18 +)	Mean RSD % (C18 −)	Global Mean RSD %
PLASMA	MeOH	30	24	27	19	**25**
	MeOH:ACN	21	32	20	24	**24**
	ACN	26	37	22	68	**38**
	Hybrid-SPE/ACN	44	46	30	35	**39**
	Hybrid-SPE/MeOH	51	46	43	38	**45**
SERUM	MeOH	30	43	27	19	**30**
	MeOH:ACN	36	41	24	39	**35**
	ACN	36	80	43	74	**58**
	Hybrid-SPE/ACN	45	38	26	56	**41**
	Hybrid-SPE/MeOH	47	100	47	50	**61**

The table presents the average RSD (%) computed for all potential metabolite features identified in plasma and serum through the use of five extraction techniques. The RSD for each potential metabolite feature was determined by examining the raw signal intensities from extraction replicates (*n* = 3). The number of features with RSD ≤ 30% (between replicates) represents high quality features that could be applied for identifying biomarkers and conducting pathway analysis in global metabolomics studies. *MeOH* methanol, *MeOH:ACN* methanol combined with acetonitrile, *ACN* acetonitrile, *SPE* solid preparation extraction, *RSD* relative standard deviation

#### Complementarity

**Fig. 3:**
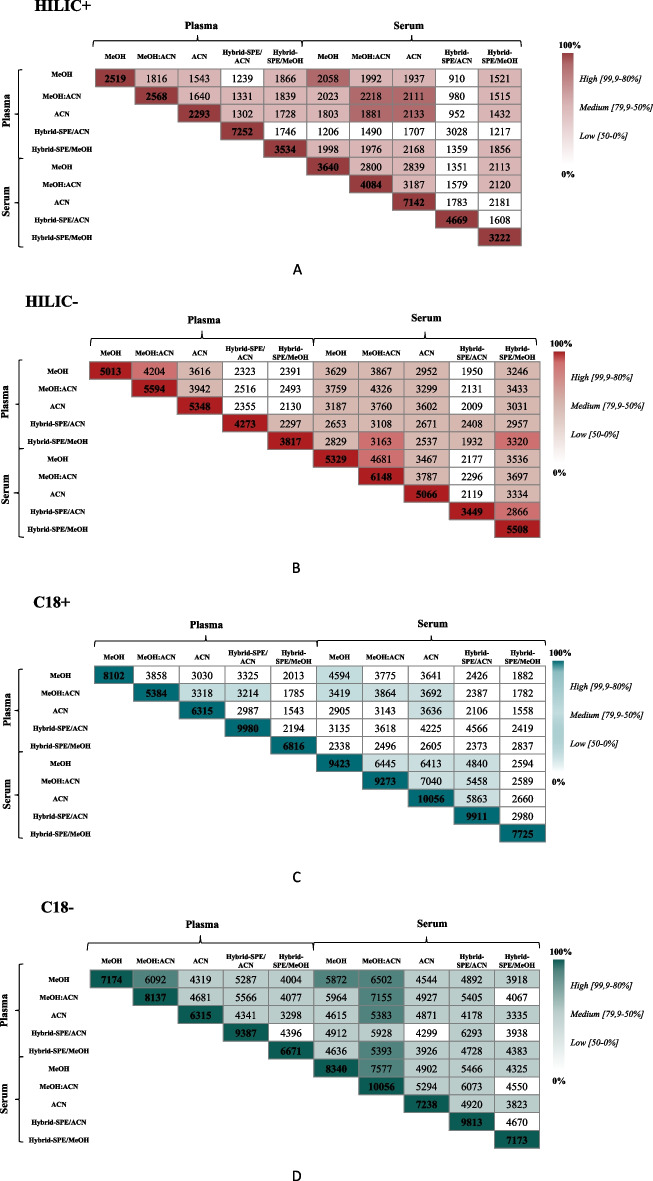
Pairwise overlap coverage of plasma vs. serum according to different extraction methods. Samples are analyzed using (i) + ESI HILIC, (ii) − ESI HILIC, (iii) + ESI RP C18 and, (iv) − ESI RP C18. The boxes with dark color and bolded numbers along the diagonal indicate the total count of potential metabolites detected using that extraction method on either plasma or serum. The gradient colors indicate high (99.9–80%), medium (79.9–50.0%), or low (50.0–0%) similarity of the potential metabolite populations seen by the two extraction methods specified. Thus, the methods indicated by the light-colored boxes are the extraction methods with the greatest overlap among all five extraction methods evaluated. (According to [6]). Each panel represents the results from a particular LC technology combined to a particular electrospray polarity setting: **A**: HILIC Column with ESI+, **B**: HILIC Column with ESI-, **C**: C18 column with ESI+, **D**: C18 column with ESI-. *MeOH* methanol, *MeOH:ACN* methanol combined with acetonitrile, *ACN* acetonitrile, *SPE* solid preparation extraction

Finally, cross-overlap analysis was conducted to determine the pairwise overlap for each of the tested methods. The results, shown in Fig. 3 and in the Additional file 9: Table S5, indicate a moderate to high degree of overlap in the metabolites detected between plasma and serum samples that were extracted using the same method and analyzed with the same LC–MS protocol. We also demonstrated that in general there was a moderate to high redundancy between the three conventional extraction methods and the two hybrid SPE, respectively. Interestingly, coverage between conventional and hybrid methods for a given sample type/LC–MS method showed a potential for complementarity (Fig. 3; Additional file 9: Table S5).

### Targeted metabolomics analysis

The use of stable isotopically unlabelled and labelled standards is largely validated in metabolomics studies. These internal standards have become essential to assess system stability, method development or data quality [21]. Herein, the list of standards was generated using the features provided by the CD3.3 software, including name, mass weigh expected and retention time (as shown in Additional file 10: Table S6). In the context of our study, the use of standards with known concentrations provided additional reliability to our bioanalytical methods. A commercial cocktail (MII) was used to provide a wide distribution of molecule with various characteristics (glucides, amino acids, etc.) to permit us to properly assess the extraction and analyses methods.

### Unlabelled analytes

#### Extraction

Additional file 11: Table S7 depicts the number of unlabelled analytes identified, following each extraction protocols for each LCMS analysis. Small differences in the extractability between plasma and serum were observed, with plasma allowing the extraction of a larger amount of standard. Within each LCMS analysis, extraction methods exhibited similar extractability (Additional file 11: Table S7). The heatmap also showed that responses for most of analytes were the same with approximatively 10^7^ in relative abundance. Only two analytes, ketoleucine and phthalic acid, were detected under specific-ESI HILIC conditions at 10^9^ (Additional file 4: Fig. S4).

#### Repeatability

**Fig. 4:**
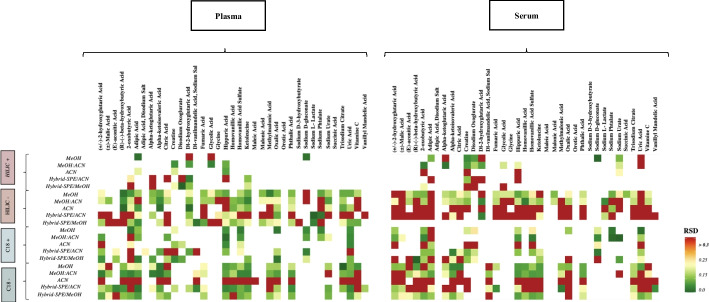
Heatmaps reflecting repeatability of standard unlabeled analytes from plasma (left) and serum (right). Heatmap, arranged by five extraction methods (MeOH, MeOH:ACN, ACN, Hybrid-SPE/ACN, and Hybrid-SPE/MeOH) within four LC–MS analysis (+ ESI HILIC, − ESI HILIC, + ESI RP C18, and − ESI RP C18), comparing the mean value (*n* = 3) repeatability (RSD) for each analytes. RSD value of analyte over 0.3 is considered as no-interpretable. Colour coding: RSD increases from dark green to dark red; White box: unidentified standard. *MeOH* methanol, *MeOH:ACN* methanol combined with acetonitrile, *ACN* acetonitrile, *SPE* solid preparation extraction, *ESI* electrospray ionisation, *RP* reverse phase, *RSD* relative standard deviation

Further efforts were focused on the precision of these unlabelled analytes. The RSD (Additional file 12: Table S8) showed similar trends to what was shown for untargeted approach (Table 1). Methanol and methanol/acetonitrile methods were outperforming both acetonitrile and hybrid SPE methods with still higher performance for plasma versus serum as a matrix. A visual representation by heatmap was used to show the extraction precision for each unlabelled analytes detected in within each LCMS analysis (Fig. 4). Above the threshold of 30% (or 0.3), the box of the concerned analyte is necessarily red. The results were more explicit in the − ESI HILIC and − ESI RP C18 analyses, due to a higher number of analytes detected. For instance, acetonitrile and hybrid-SPE with acetonitrile methods in − ESI HILIC analysis provided acceptable precision (≤ 30% RSD) for only five standards (alpha-ketoisovaleric acid, dl 2-hydroxyglutaric acid, ketoleucine, sodium l-lactacte and sodium phtalate).

### Labelled analytes

#### Extraction

The finding was also the same as before. All the results for labelled analytes can be found in Additional file 11: Table S7.

#### Repeatability

Similar precision approach was also realized for labelled analytes (Additional file 13: Table S9). The results demonstrated a similar trend related to global reproducibility (Table 2). Only methanol and methanol/acetonitrile in plasma obtained RSD ≤ 30% demonstrating a good repeatability and reproducibility. This corroborates with unlabelled analytes results. However, for the remaining plasmatic extraction methods and the whole of serum extraction methods, reproducibility was very poor with RSD significantly higher to 30%. Such data suggest that care must be taken in the choice of matrix and extraction method.

**Table 2:** Summary of total number of labeled standard analytes which experienced matrix effect (suppression or enhancement) from plasma vs. serum

	HILIC					C18				
	MeOH	MeOH:ACN	ACN	Hybrid-SPE/ACN	Hybrid-SPE/MeOH	MeOH	MeOH:ACN	ACN	Hybrid-SPE/ACN	Hybrid-SPE/MeOH
Plasma										
Suppressed (+ESI)	2	3	0	1	2	0	0	0	0	1
Enhanced (+ESI)	1	0	3	0	0	4	2	3	4	2
Total affected (+ESI)	3	3	3	1	2	4	2	3	0	3
Total unaffected (+ESI)	0	0	0	1	1	0	1	0	0	1
Suppressed (− ESI)	3	0	0	1	6	1	0	1	0	1
Enhanced (− ESI)	1	2	5	3	0	0	2	1	3	1
Total affected (− ESI)	4	2	5	4	6	1	2	2	3	2
Total unaffected (− ESI)	2	3	0	2	0	3	2	0	0	2
Serum										
Suppressed (+ESI)	0	0	1	0	2	1	0	0	0	3
Enhanced (+ESI)	1	1	1	3	0	1	4	4	1	1
Total affected (+ESI)	1	1	2	3	2	2	4	4	1	4
Total unaffected (+ESI)	2	1	1	0	0	2	0	0	0	0
Suppressed (− ESI)	1	0	0	3	2	2	1	1	0	2
Enhanced (− ESI)	5	6	6	3	0	1	2	2	3	0
Total affected (− -ESI)	6	6	6	6	2	3	3	3	3	2
Total unaffected (− ESI)	1	0	0	0	0	1	0	0	0	1

Table shows matrix effect of plasma vs. serum across five extraction methods (MeOH, MeOH:ACN, ACN, Hybrid-SPE/ACN, and Hybrid-SPE/MeOH) within four LC–MS analysis (+ ESI HILIC, − ESI HILIC, + ESI RP C18, and − ESI RP C18). An analyte is considered to have been affected if its matrix effect ratio is above 120% or suppressed if it is below 80%. (According to [6]). *MeOH* methanol, *MeOH:ACN* methanol combined with acetonitrile, *ACN* acetonitrile, *SPE* solid preparation extraction, *ESI* electrospray ionisation, *RP* reverse phase

#### Linearity and sensitivity

**Fig. 5:**
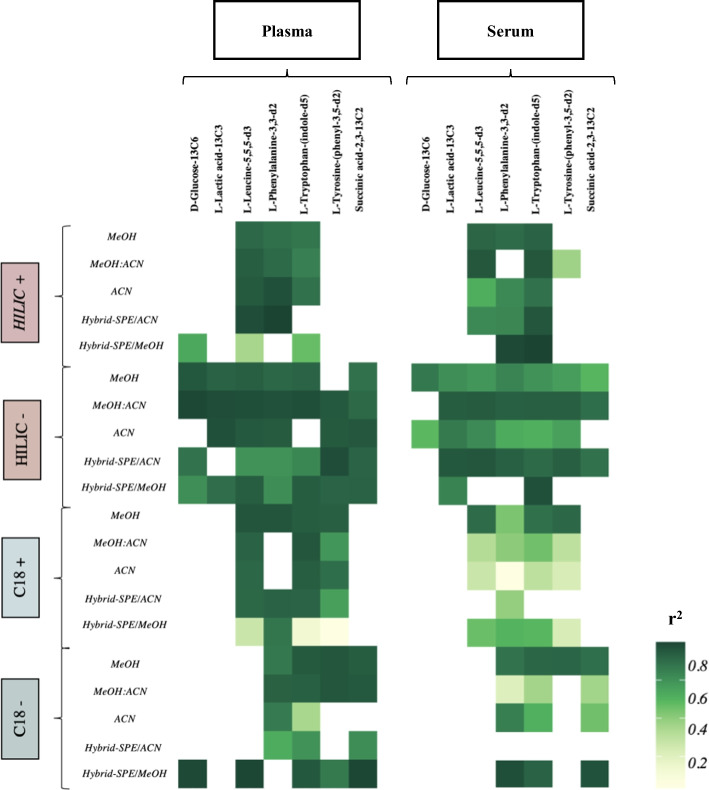
Heatmaps reflecting linearity (*r*^2^) of standard unlabeled analytes from plasma (left) and serum (right). Heatmap, arranged by five extraction methods (MeOH, MeOH:ACN, ACN, Hybrid-SPE/ACN, and Hybrid-SPE/MeOH) within four LC–MS analysis (+ ESI HILIC, − ESI HILIC, + ESI RP C18, and − ESI RP C18), comparing the mean value (*n* = 3) linearity (*r*^2^) for each analytes. *r*^2^ (or coefficient of determination) value should be as close to 1 as possible. Colour coding: r^2^ increases from light to dark green; White box: unidentified standard. *MeOH* methanol, *MeOH:ACN* methanol combined with acetonitrile, *ACN* acetonitrile, *SPE* solid preparation extraction, *ESI* electrospray ionisation, *RP* reverse phase

Due to the different concentration ranges of the analytes in the plasma and serum samples, it is essential that the analytical methods cover a large dynamic range, and that they provide sufficient sensitivity to permit the quantification of molecules at both low and high concentrations [22]. To assess these parameters, increasing amounts of labelled analytes were added before sample preparation, and linear calibration curves were calculated. The coefficient of determination (*r*^2^) was calculated for all analytes in each extraction method and represented by heatmap with colour gradient of *r*^2^ intensity (Fig. 5). Moreover, the mean *r*^2^ for each extraction across all LC–MC methods was also determined in Additional file 14: Table S10. Our results demonstrate that the methanol and methanol/acetonitrile methods obtained the highest linear calibration curves (*r*^2^ ≥ 0.9). Sensitivity was also provided by the calibration curve slope, and the LOD and LOQ values were calculated as those corresponding to the signal-to-noise ratios of 3:1 and 10:1, respectively. The lowest LOD and LOQ values were obtained for l-Tyrosine-(phenyl-3,5-d2) (LOD 0.02 μM, LOQ 0.06 μM), and the highest values corresponded to l-Phenylalanine-3,3-d2 (LOD 19.5 μM, LOQ 59 μM). The quantification of these limits showed that the hybrid-SPE with acetonitrile method was the most performant to determine the metabolite compounds in plasma samples (Additional file 15: Table S11).

#### Matrix effects

These were evaluated to a middle concentration level. All analytes were overall impacted by ionization suppression and enhancement with greater matrix effects in serum (Additional file 16: S12; Table 2). Comparing each LC–MS methods, a minimization of matrix effects was observed in methanol and methanol/acetonitrile methods, and again confirms their good extraction performance.

## Discussion

To our knowledge, this is the first study to validate such a diverse range of extraction and liquid-chromatography (LC) methods for both plasma and serum samples.

### Untargeted (global) metabolomics analysis

By comparing the results from electrophoresis and SDS-PAGE analysis, it must be pointed out that interferences between methanol solvent and SPE column resulted to incomplete elution. Nevertheless, considering that the total protein concentration in plasma or serum is approximately 70,000 µg/mL [20], all extraction protocols worked properly but care must be taked when using hybrid-SPE, particulary for analyses with high sensitivity to protein interference.

As for metabolomics coverage, results were in line with previous studies, which however were limited to plasma explorations [23]. A limitation apparent, also found in many studies, is the high proportion of putative metabolites detected. Despite the precision of our experiment, a limited number of samples could be the reason but for the rest of the study, this consequence was not a bias. Concerning *“biologically meaningless noise level”* for − ESI HILIC analysis, this phenomenon was totally independent from extraction because this method is known to the high noise level [24, 25].

The reproducibility analysis showed that a significant portion of features were not reproducible (i.e., RSD > 30%) in serum methods, indicating that the use of these methods would necessitate the implementation of additional quality controls and a thorough examination of the causes of the irreproducibility. Regarding the acetonitrile precipitation method, previous studies for plasma or serum metabolomics produced conflicting evidence regarding repeatability, however, none of these studies used RSD as precision parameter, limiting their results to signal to noise (S/N) ratio or chromatogram observation [26, 27]. For hybrid-SPE methods, it is difficult to explain such results in the absence of other comparative studies. Considering this high RSD was found for both SPE methods in either plasma or serum, our results highlight the importance of carefully controlling SPE-based protocols when used routinely.

The cross-overlap findings are in accordance with findings reported by more targeted studies, that highlighted this strong overlap in detected metabolites between plasma and serum matrices [21]. Regarding the complementarity between extraction methods for a given sample type/LC–MS method, it suggests that extraction methods could offer a complementarity towards providing as complete as possible an image of the metabolic landscape. As demonstrated by Tulipani et al., conventional and hybrid-SPE methods may fruitfully cohabit in untargeted metabolomics protocols [13]. However, this needs to be carefully considered in regard to the performance of each method Since deploying these methods involves a sample consumption, a fivefold increase in MS analysis time and the use of hybrid-SPE, with reproducibility issues as demonstrated above, the potential gain in coverage needs to be evaluated with attention. For instance, in + ESI HILIC analysis, a total of 6286 non-redundant putative metabolites were detected in the five combined plasma and serum extraction methods. At this stage of the study, we believe the most performant methods are methanol and methanol/acetonitrile. Consequently, in + ESI HILIC analysis, this represented only a 40% improvement over methanol or methanol/acetonitrile in plasma and 60% improvement of methanol or methanol/acetonitrile in serum. The approach was realised in the other 3 conditions and similar results were observed. The improvement was approximatively 50% (− ESI HILIC plasma), 49% (− ESI HILIC serum), 35% (+ ESI RP plasma), 64% (+ ESI RP serum), 74% (− ESI RP plasma), and 75% (− ESI RP serum). Therefore, these results clearly showed that simply using multiple extraction methods in parallel could not be the best way to increase metabolite coverage.

### Targeted metabolomics analysis

The comparison of standards across different extraction methods was achievable because the same amount of sample was used for all protocols, the same dilution was applied prior to injection, and the same MS detection parameters were employed, allowing for comparable peak areas. In agreement with prior studies, our results showed that the extraction methods did not appear to affect peak area [28].

A similar pattern of results was obtained in as for reproducibility in targeted approach. Sitnikov et al. also observed a better RDS in conventional method, notably methanol-based method than hybrid-SPE methods [9]. However, incoherence could be demonstrated, as the Michopoulos et al. study who highlighted an RSD of 20% for hybrid-SPE method compared to 46% for methanol-precipitated methods [29]. We acknowledge that there is considerable variability among techniques. We speculate that this might be due to the use of different SPE column suppliers or a different selection of sorbent characteristics and wash/elution conditions. Further investigations could be realized.

Pooled biological plasma and serum samples were used to avoid biological variability in the comparative analysis among sample preparation procedures. The majority of results demonstrated a superiority of plasma *vs.* serum. This could be explained by the waiting time at room temperature during serum separation from whole blood. To date, temperature is known to induce significant changes in the metabolite performance and affect extraction, robustness, sensitivity, or matrix effect [30].

Regarding the extraction methods, studies support the notion that hybrid-SPE could be considered as the best method. It should be noted that these studies were focused on targeted metabolomics approaches with SPE columns from another supplier, different solid and mobile phases, and which affects ionisation and therefore results [23]. Consequently, true comparison of SPE with classical approaches is difficult to draw out at this point. In contrast, even if some of our results differed from prior studies, the conclusion that methanol and methanol/acetonitrile methods for plasma seemed to be the most efficient, is supported by the coherence of these experiments.

## Conclusion

**Fig. 6:**
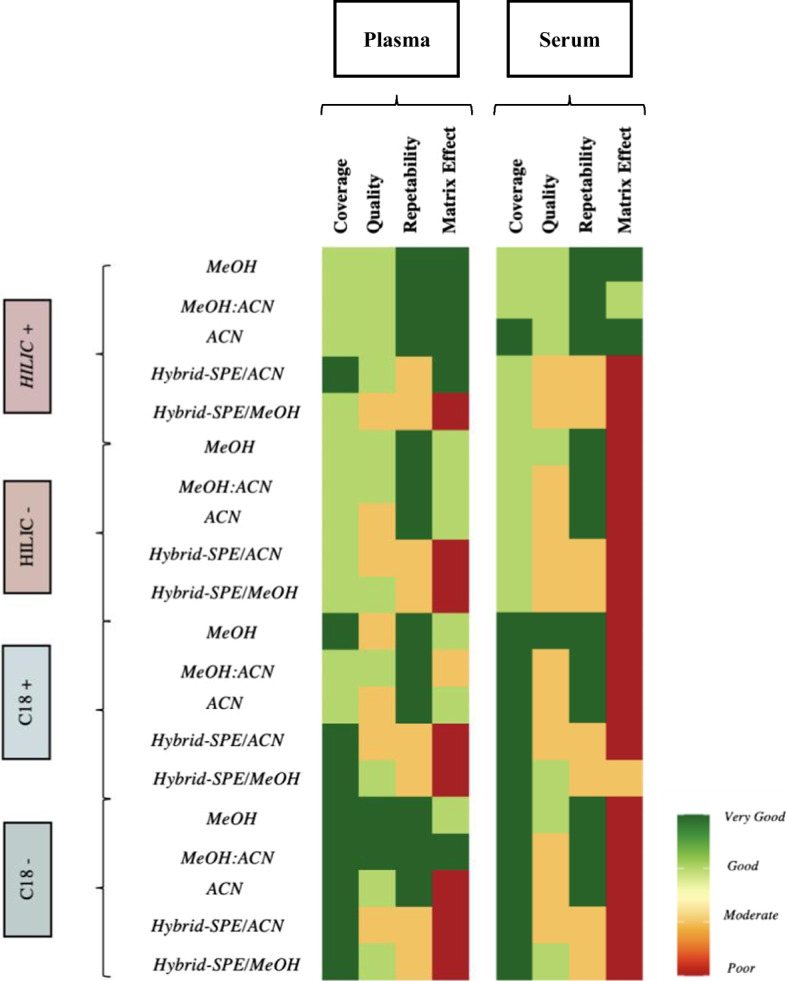
Summary of extract method performance for plasma vs. Serum. Color coding: the scoring of method performance where dark red is the worst and dark green is the best. *MeOH* methanol, *MeOH:ACN* methanol combined with acetonitrile, *ACN* acetonitrile, *SPE* solid preparation extraction, *ESI* electrospray ionisation, *RP* reverse phase

A showdown between five extraction methods for plasma vs. serum metabolomics analysis was realized. For the first time, coverage, repeatability, overlap and matrix effect were systematically assessed and compared in combination with two reversed-phased and two HILIC methods. Our results confirm the superiority of methanol-based precipitation methods vs. other methods, with the best results observed using methanol or methanol/acetonitrile as shown in Fig. 6. We also reveal high overlap of methanol-based methods and hybrid-SPE to each other, providing the possibility of increased metabolome coverage, however we highlight that such potential benefits must be weighed against time constrains and the risk of low reproducibility of hybrid-SPE method. Despite certain contradiction with prior studies, these approaches are invaluable in this newly emerging field. Furthermore, we also highlighted the careful consideration about matrix choice. Plasma showed the most suitable in this metabolomics approach combined with methanol-based methods. While further investigations could be necessary to validate the some of our observations, we hope they supply some guideline in designing metabolomics protocols and highlights the considerations to be considered.

## Supplementary Information

**Additional file 1. Figure S1**. Study design for the targeted and global metabolomics analysis of extraction methods. Biological samples and spiked solvents are simultaneously prepared. From stock solutions, MI and MII are diluted at different concentrations in five solvents. Plasma and serum samples are prepared from pooled of 7 donors. Five extractions methods are applied in both plama and serum matrices in triplicate. For each method, we generatetwelve plasma and serum extracts spiked with MI,three plasma and serum extracts spiked with MII,three plasma and serum extracts unspiked, andsix neat solventsspiked with MII. The next of protocol is described in materiel and methods part. MeOH : methanol; MeOH:ACN : methanol combined with acetonitrile; ACN: acetonitrile.

**Additional file 2**: **Figure S2**. Customs parameters used for data processing with Compound Discoverer software.

**Additional file 3: Figure S3**. Quality analysis of plasmatic and serum extracts by SDS PAGE SDS-PAGE of loaded and eluted plasma and serum proteins, after five different deproteinizations. 1: Molecular Weight marker; 2: neat MeOH; 3: Plasma extract with MeOH; 4: neat MeOH:ACN; 5: Plasma extract MeOH:ACN; 6: neat ACN, 7: Plasma extract with ACN, 8: neat hybrid-SPE with ACN, 9: Plasma extract with hybrid-SPE with ACN; 10: neat hybrid-SPE with MeOH; 11: Plasma extract with hybrid-SPE with MeOH; 12: Molecular Weight marker; 13: neat MeOH; 14: Serum extract with MeOH; 15: neat MeOH:ACN; 16: Serum extract MeOH:ACN; 17: neat ACN, 18: Serum extract with ACN, 19: neat hybrid-SPE with ACN, 20: Serum extract with hybrid-SPE with ACN; 21: neat hybrid-SPE with MeOH; 22: Serum extract with hybrid-SPE with MeOH.

**Additional file 4: Figure S4**. Heatmaps reflecting the peak area of standard unlabeled analytes from plasmaand serumHeatmap, reflecting the response of the detected unlabeled standards from plasmavs. serumunder five extraction methodswithin four LC-MS analysis. Colour coding: signal responseincreases from light to dark green ; White box : unidentified standard MeOH : methanol; MeOH:ACN : methanol combined with acetonitrile; ACN: acetonitrile; SPE : solid preparation extraction; ESI : electrospray ionisation; RP: reverse phase.

**Additional file 5: Table S1**. List of isotopic unlabeled and labeled standard analytes. Following information include specifications, stock solution concentrations stored at -80°C, and final solution preparations for each standards. Different final concentrationsare diluted in five solvents, acetonitrile, 0,1% formic acid/methanol, and 0,1% formic acid/acetonitrile) for each standards. A calibration curve is then created for the assessment of linearity, sensitivity and matrix effects. MI : 7 labeled standard mixed; MII : unlabeled organic acid commercial mix.

**Additional file 6. Table S2**. List of compound expected containing name, formula and molecular weight of standards imported in search mass lists parameter from CD3.3.

**Additional file 7. Table S3**. Quality analysis of plasmatic and serum extracts by BCA assayThis table shows the protein concentrationin neat solventsand after plasma / serum extractions by BCA assay. MeOH : methanol; MeOH:ACN : methanol combined with acetonitrile; ACN: acetonitrile, SPE : solid preparation extraction.

**Additional file 8. Table S4**. Ratio of remaining proteins to total proteins in plasma and serum for all extraction methods. The remaining protein results were obtained after BCA assay and the values were in Table I. Total proteins in plasma and serum refer to concentration of 70.000 ug/mL. MeOH : methanol; MeOH:ACN : methanol combined with acetonitrile; ACN: acetonitrile; SPE : solid preparation extraction; ESI : electrospray ionisation; RP: reverse phase.

**Additional file 9. Table S5**. Overlap coverage between plasma and serum using the same method and analysed with the same LC-MS protocol.MeOH : methanol; MeOH:ACN : methanol combined with acetonitrile; ACN: acetonitrile; SPE : solid preparation extraction; ESI : electrospray ionisation; RP: reverse phase.

**Additional file 10. Table S6**. Unlabeled and labeled standard analytes used in the study. Mass weight expected and retention times are obtained from CD3.3 with several database onlines. Retention times are analyzed in either ESI mode of each column. ND stands for not detected. For details on the usage and fate of analytes in experiments see next Supplementary Tables and Figures. ESI : electrospray ionisation; RP: reverse phase.

Additional file 11. Table S7. Number of unlabeled and labeled detected standard in different protocols. All analytes are detected from CD3.3.MeOH : methanol; MeOH:ACN : methanol combined with acetonitrile; ACN: acetonitrile; SPE : solid preparation extraction; ESI : electrospray ionisation; RP: reverse phase.

**Additional file 12. Table S8**. Summary of repeatabilityfor unlabeled standard analytes across all extraction methods and LC-MS analyses. The table displays a mean relative standard deviationfor plasma vs. serum in each LC-MS conditions . Empty boxes stands not detected.MeOH : methanol; MeOH:ACN : methanol combined with acetonitrile; ACN: acetonitrile; SPE : solid preparation extraction; ESI : electrospray ionisation; RP: reverse phase.

**Additional file 13. Table S9**. Summary of repeatabilityfor labeled standard analytes across all extraction methods and LC-MS analyses. The table displays a mean relative standard deviationfor plasma vs. serum in each LC-MS conditions . Empty boxes stands not detected.MeOH : methanol; MeOH:ACN : methanol combined with acetonitrile; ACN: acetonitrile; SPE : solid preparation extraction; ESI : electrospray ionisation; RP: reverse phase.

**Additional file 14. Table S10**. Summary of linearityfor labeled standard analytes across all extraction methods and LC-MS analyses. The table displays a mean r2for plasma vs. serum in each LC-MS conditions . Empty boxes stands not detected. MeOH : methanol; MeOH:ACN : methanol combined with acetonitrile; ACN: acetonitrile; SPE : solid preparation extraction; ESI : electrospray ionisation; RP: reverse phase.

**Additional file 15. Table S11**. Summary of sensitivityfor labeled standard analytes across all extraction methods and LC-MS analyses. Sensitivity is given by the calibration curve slope, and the LOD and LOQ values are calculated as those corresponding to the signal-to-noise ratios of 3:1 and 10:1, respectively. The table displays a mean of LOD and LOQfor plasma vs. serum in each LC-MS conditions. Empty boxes stands not detected. LOD : limit of detection; LOQ : limit of quantitation; MeOH : methanol; MeOH:ACN : methanol combined with acetonitrile; ACN: acetonitrile; SPE : solid preparation extraction; ESI : electrospray ionisation; RP: reverse phase.

**Additional file 16. Table S12**. Summary of matrix observed effects observed for labeled standard analytes across all extraction methods and LC-MS analyses. Not all analytes were successfully detected in plasma or serum. Matrix effect in the range of 0.80 < ME < 1.20 are considered as negligible and labelled as “no matrix effect”. ME values below 0.80 were considered as ion suppression, and those above 1.20 as ion enhancement. Empty boxes stands not detected. MeOH : methanol; MeOH:ACN : methanol combined with acetonitrile; ACN: acetonitrile; SPE : solid preparation extraction; ESI : electrospray ionisation; RP: reverse phase.

**Additional file 17. Table S13**: Number of putative metabolites detected and mean RSD in different protocols with ratio 1:3.

**Additional file 18. Table S14**: Unlabeled and labeled standard analytes used in the study. Mass weight expected, retention times and m/z are obtained from CD3.3 with several database onlines. MeOH : methanol; MeOH:ACN : methanol combined with acetonitrile; ACN: acetonitrile; SPE : solid preparation extraction.

**Additional file 19. Table S15**: Plasma chromatograms highlighting the differences between sample preparation protocols.The chromatograms were obtained using the Freestyle software. MeOH : methanol; MeOH:ACN : methanol combined with acetonitrile; ACN: acetonitrile; SPE : solid preparation extraction.

## Abbreviations

LC–MS: Liquid chromatography coupled with mass spectrometry
MeOH: Methanol
ACN: Acetonitrile
SPE: Solid phase extraction
RP: Reverse-phase
HILIC: Hydrophilic interaction chromatography
RSD: Relative standard deviation
ME: Matrix effect

## Declarations

### Availability of data and materials

Data will be made available on request.

## References

[1] Rahbaran M, Zekiy AO, Bahramali M, Jahangir M, Mardasi M, Sakhaei D (2022). Therapeutic utility of mesenchymal stromal cell (MSC)-based approaches in chronic neurodegeneration: a glimpse into underlying mechanisms, current status, and prospects. Cell Mol Biol Lett..

[2] Ashrafian H, Sounderajah V, Glen R, Ebbels T, Blaise BJ, Kalra D (2021). Metabolomics: the stethoscope for the twenty-first century. MPP.

[3] Keshavarz M, Solaymani-Mohammadi F, Namdari H, Arjeini Y, Mousavi MJ, Rezaei F (2020). Metabolic host response and therapeutic approaches to influenza infection. Cell Mol Biol Lett.

[4] Liang B, Burley G, Lin S, Shi YC (2022). Osteoporosis pathogenesis and treatment: existing and emerging avenues. Cell Mol Biol Lett..

[5] Johnson CH, Ivanisevic J, Siuzdak G (2016). Metabolomics: beyond biomarkers and towards mechanisms. Nat Rev Mol Cell Biol.

[6] Clish CB (2015). Metabolomics: an emerging but powerful tool for precision medicine. Cold Spring Harb Mol Case Stud..

[7] Mannello F (2008). Serum or plasma samples? The « Cinderella » role of blood collection procedures: preanalytical methodological issues influence the release and activity of circulating matrix metalloproteinases and their tissue inhibitors, hampering diagnostic trueness and leading to misinterpretation. Arterioscler Thromb Vasc Biol avr.

[8] Yu Z, Kastenmüller G, He Y, Belcredi P, Möller G, Prehn C (2011). Differences between human plasma and serum metabolite profiles. PLoS ONE.

[9] Sitnikov DG, Monnin CS, Vuckovic D (2016). Systematic assessment of seven solvent and solid-phase extraction methods for metabolomics analysis of human plasma by LC-MS. Sci Rep.

[10] Kind T, Fiehn O (2010). Advances in structure elucidation of small molecules using mass spectrometry. Bioanal Rev..

[11] Tulipani S, Llorach R, Urpi-Sarda M, Andres-Lacueva C (2013). Comparative analysis of sample preparation methods to handle the complexity of the blood fluid metabolome: when less is more. Anal Chem.

[12] Alshammari TM, Al-Hassan AA, Hadda TB, Aljofan M (2015). Comparison of different serum sample extraction methods and their suitability for mass spectrometry analysis. Saudi Pharmaceut J..

[13] Tulipani S, Mora-Cubillos X, Jáuregui O, Llorach R, García-Fuentes E, Tinahones FJ (2015). New and vintage solutions to enhance the plasma metabolome coverage by LC-ESI-MS untargeted metabolomics: the not-so-simple process of method performance evaluation. Anal Chem.

[14] Medina J, van der Velpen V, Teav T, Guitton Y, Gallart-Ayala H, Ivanisevic J (2020). Single-step extraction coupled with targeted HILIC-MS/MS approach for comprehensive analysis of human plasma lipidome and polar metabolome. Metabolites.

[15] Höring M, Stieglmeier C, Schnabel K, Hallmark T, Ekroos K, Burkhardt R (2022). Benchmarking one-phase lipid extractions for plasma lipidomics. Anal Chem.

[16] Defossez E, Bourquin J, von Reuss S, Rasmann S, Glauser G (2023). Eight key rules for successful data-dependent acquisition in mass spectrometry-based metabolomics. Mass Spectrom Rev.

[17] Dunn WB, Broadhurst D, Begley P, Zelena E, Francis-McIntyre S, Anderson N (2011). Procedures for large-scale metabolic profiling of serum and plasma using gas chromatography and liquid chromatography coupled to mass spectrometry. Nat Protoc.

[18] 5.4 Quantitative estimation of matrix effect, recovery and process efficiency [Internet]. [cité 21 déc 2022]. Disponible sur: https://sisu.ut.ee/lcms_method_validation/54-quantitative-estimation-matrix-effect-recovery-process-efficiency.

[19] Guo Y (2015). Recent progress in the fundamental understanding of hydrophilic interaction chromatography (HILIC). Analyst.

[20] Leeman M, Choi J, Hansson S, Storm MU, Nilsson L (2018). Proteins and antibodies in serum, plasma, and whole blood—size characterization using asymmetrical flow field-flow fractionation (AF4). Anal Bioanal Chem.

[21] Broadhurst D, Goodacre R, Reinke SN, Kuligowski J, Wilson ID, Lewis MR (2018). Guidelines and considerations for the use of system suitability and quality control samples in mass spectrometry assays applied in untargeted clinical metabolomic studies. Metabolomics.

[22] Moosavi SM, Ghassabian S, Moosavi SM, Ghassabian S. Linearity of calibration curves for analytical methods: a review of criteria for assessment of method reliability [Internet]. Calibration and validation of analytical methods—a sampling of current approaches. IntechOpen; 2018 [cité 21 déc 2022]. Disponible sur: https://www.intechopen.com/state.item.id.

[23] Contrepois K, Jiang L, Snyder M (2015). Optimized analytical procedures for the untargeted metabolomic profiling of human urine and plasma by combining hydrophilic interaction (HILIC) and reverse-phase liquid chromatography (RPLC)-mass spectrometry. Mol Cell Proteomics.

[24] Periat A, Boccard J, Veuthey JL, Rudaz S, Guillarme D (2013). Systematic comparison of sensitivity between hydrophilic interaction liquid chromatography and reversed phase liquid chromatography coupled with mass spectrometry. J Chromatogr A.

[25] Hosseinkhani F, Huang L, Dubbelman AC, Guled F, Harms AC, Hankemeier T (2022). Systematic evaluation of HILIC stationary phases for global metabolomics of human plasma. Metabolites.

[26] Wawrzyniak R, Kosnowska A, Macioszek S, Bartoszewski R, Markuszewski M (2018). New plasma preparation approach to enrich metabolome coverage in untargeted metabolomics: plasma protein bound hydrophobic metabolite release with proteinase K. Sci Rep.

[27] Pires FAR, Ramalhete LM, Ribeiro E, Calado CRC. Impact of the solvent extraction method on the plasma metabolome profile. In: 2019 IEEE 6th Portuguese Meeting on Bioengineering (ENBENG). 2019. p. 1–4.

[28] Reis A, Rudnitskaya A, Blackburn GJ, Mohd Fauzi N, Pitt AR, Spickett CM (2013). A comparison of five lipid extraction solvent systems for lipidomic studies of human LDL. J Lipid Res.

[29] Michopoulos F, Lai L, Gika H, Theodoridis G, Wilson I (2009). UPLC-MS-based analysis of human plasma for metabonomics using solvent precipitation or solid phase extraction. J Proteome Res.

[30] Stevens VL, Hoover E, Wang Y, Zanetti KA (2019). Pre-analytical factors that affect metabolite stability in human urine, plasma, and serum: a review. Metabolites.

